# Lateral episiotomy or no episiotomy in vacuum assisted delivery in nulliparous women (EVA): multicentre, open label, randomised controlled trial

**DOI:** 10.1136/bmj-2023-079014

**Published:** 2024-06-17

**Authors:** Sandra Bergendahl, Maria Jonsson, Susanne Hesselman, Victoria Ankarcrona, Åsa Leijonhufvud, Anna-Carin Wihlbäck, Tove Wallström, Emmie Rydström, Hanna Friberg, Helena Kopp Kallner, Sophia Brismar Wendel

**Affiliations:** 1 Department of Clinical Sciences, Danderyd Hospital, Karolinska Institutet, Stockholm, Sweden; 2 Department of Women’s and Children’s Health, Uppsala University, Uppsala, Sweden; 3 Department of Women’s and Children’s Health, Centre for Clinical Research Dalarna, Falun, Sweden; 4 Department of Clinical Science Helsingborg, Lund University, Helsingborg, Sweden; 5 Department of Clinical Sciences, Obstetrics and Gynecology, Umeå University, Umeå, Sweden; 6 Department of Clinical Science and Education, South General Hospital, Karolinska Institutet, Stockholm, Sweden; 7 Department of Obstetrics and Gynecology, Växjö Central Hospital, Växjö, Sweden; 8 Department of Obstetrics and Gynecology, Sahlgrenska University Hospital, Göteborg, Sweden

## Abstract

**Objective:**

To assess the effect of lateral episiotomy, compared with no episiotomy, on obstetric anal sphincter injury in nulliparous women requiring vacuum extraction.

**Design:**

A multicentre, open label, randomised controlled trial.

**Setting:**

Eight hospitals in Sweden, 2017-23.

**Participants:**

717 nulliparous women with a single live fetus of 34 gestational weeks or more, requiring vacuum extraction were randomly assigned (1:1) to lateral episiotomy or no episiotomy using sealed opaque envelopes. Randomisation was stratified by study site.

**Intervention:**

A standardised lateral episiotomy was performed during the vacuum extraction, at crowning of the fetal head, starting 1-3 cm from the posterior fourchette, at a 60° (45-80°) angle from the midline, and 4 cm (3-5 cm) long. The comparison was no episiotomy unless considered indispensable.

**Main outcome measures:**

The primary outcome of the episiotomy in vacuum assisted delivery (EVA) trial was obstetric anal sphincter injury, clinically diagnosed by combined visual inspection and digital rectal and vaginal examination. The primary analysis used a modified intention-to-treat population that included all consenting women with attempted or successful vacuum extraction. As a result of an interim analysis at significance level P<0.01, the primary endpoint was tested at 4% significance level with accompanying 96% confidence interval (CI).

**Results:**

From 1 July 2017 to 15 February 2023, 717 women were randomly assigned: 354 (49%) to lateral episiotomy and 363 (51%) to no episiotomy. Before vacuum extraction attempt, one woman withdrew consent and 14 had a spontaneous birth, leaving 702 for the primary analysis. In the intervention group, 21 (6%) of 344 women sustained obstetric anal sphincter injury, compared with 47 (13%) of 358 women in the comparison group (P=0.002). The risk difference was −7.0% (96% CI −11.7% to −2.5%). The risk ratio adjusted for site was 0.47 (96% CI 0.23 to 0.97) and unadjusted risk ratio was 0.46 (0.28 to 0.78). No significant differences were noted between groups in postpartum pain, blood loss, neonatal outcomes, or total adverse events, but the intervention group had more wound infections and dehiscence.

**Conclusions:**

Lateral episiotomy can be recommended for nulliparous women requiring vacuum extraction to significantly reduce the risk of obstetric anal sphincter injury.

**Trial registration:**

ClinicalTrials.gov NCT02643108.

## Introduction

Obstetric anal sphincter injury is a serious complication to vaginal birth causing anal incontinence^1^ and reduced quality of life.^2^ Primiparity and instrumental birth are two major risk factors of obstetric anal sphincter injury.^3^ The obstetric anal sphincter injury rate in primiparous women varies between countries, with rates of 0.1-4% for spontaneous births and 6-24% for instrumental births in Europe, Canada, and the United States.^4^ ^5^

The preventive effect of an episiotomy, an incision made in the tissue between the vaginal opening and the anus during childbirth, on obstetric anal sphincter injury is not clear. A Cochrane review of randomised controlled trials has concluded that routine episiotomy may increase the risk of obstetric anal sphincter injury in non-instrumental birth,^6^ while lateral or mediolateral episiotomy might prevent obstetric anal sphincter injury in vacuum extraction in nulliparous women, based on results from pooled observational studies.^7^ ^8^ ^9^ ^10^ The most recent meta-analysis, published in 2022, which included 23 observational studies and two underpowered randomised controlled trials, reported an adjusted odds ratio of 0.51 (95% confidence interval (CI) 0.42 to 0.84) for obstetric anal sphincter injury when a lateral or mediolateral episiotomy was performed compared with no episiotomy.^7^ However, other observational studies reported no effect,^11^ or the opposite effect.^12^ Episiotomy has also been associated with an increased risk of postpartum haemorrhage and pain.^13^ As such, some national guidelines state that a lateral or mediolateral episiotomy should be considered in nulliparous women requiring vacuum extraction,^14^ ^15^ but also that the decision should be tailored to the circumstances,^14^ making the decision to do an episiotomy provider dependent. This uncertainty regarding treatment effect size and adverse effects is reflected in the internationally varying rates of episiotomy in instrumental births, from 17.1% in Denmark to 97.2% in Poland.^4^

Observational studies come with limitations. For instance, the differences in effect can be due to lack of standardisation regarding the type of episiotomy, where the angle and incision point have been deemed the most important traits.^16^ ^17^ ^18^ To date, no adequately sized randomised controlled trial on the protective effect of episiotomy on obstetric anal sphincter injury in vacuum extraction has been published.^19^ ^20^ Hence, the Cochrane Collaboration and the National Institute for Health and Care Excellence’s Evidence Search, among others, have stated that the protective effect of lateral or mediolateral episiotomy in vacuum extraction should be investigated in an adequately sized randomised controlled trial.^6^ ^8^ ^13^ Accordingly, we hypothesised that a routine lateral episiotomy reduces the risk of obstetric anal sphincter injury in nulliparous women requiring vacuum extraction. We performed a randomised controlled trial to assess the effect of lateral episiotomy compared with no episiotomy on obstetric anal sphincter injury in nulliparous women requiring vacuum extraction.

## Methods

### Study design

The Episiotomy in Vacuum Assisted delivery (EVA) trial was a randomised, parallel, open label, controlled trial comparing the effect of lateral episiotomy with no episiotomy (1:1), on obstetric anal sphincter injury in nulliparous women requiring vacuum extraction. Eight hospitals in Sweden conducted the study between 1 July 2017 and 15 February 2023. The study protocol has been published previously.^21^ The trial was approved by the regional ethical review board of Stockholm before the start (2015/1238-31/2) with amendments to approve additional participating hospitals (2017/1005-32, 2018/775-32, 2018/2291-32, 2019-02758, 2019-02758, and 2019-04427) and retrieval of data from the Swedish pregnancy register and the Swedish neonatal quality register (2023-02301-02). The trial was monitored by the Karolinska Trial Alliance 2017-20 and by two independent monitors 2021-23. The trial was registered in www.clinicaltrials.gov (NCT02643108) on 30 December 2015.

Results are reported according to CONSORT 2010 guidelines^22^ and TIDieR checklist.^23^

### Participants

Inclusion criteria were nulliparous women with a singleton, live, cephalic presenting fetus at 34 gestational weeks or more, requiring vacuum extraction. Exclusion criteria were previous surgery for urinary or anal incontinence or for genital prolapse. Women here refers to people of female sex. All genders were eligible. Informed written and oral consent was obtained by attending midwives or physicians after gestational week 18, including during labour if the woman had adequate pain relief and time to reflect, based on the healthcare provider’s judgement.

### Randomisation and masking

The decision to perform a vacuum extraction was made by the attending physician on obstetric indications independent of trial participation. This instruction was clearly made to physicians during trial education and to patients in the consent form, to avoid biased trial inclusion. Randomisation took place after the decision. The randomisation sequence was produced by Karolinska Trial Alliance using computer-based random permuted blocks of two to eight stratified by site. The allocation was done at a 1:1 ratio using consecutive opaque sealed envelopes to facilitate inclusion in medically urgent situations. After informed consent was confirmed, the sealed envelope was opened by the assisting nurse, midwife, or physician, and the allocation was read out loud in the delivery room. No masking was possible.

### Procedures

**Fig 1:**
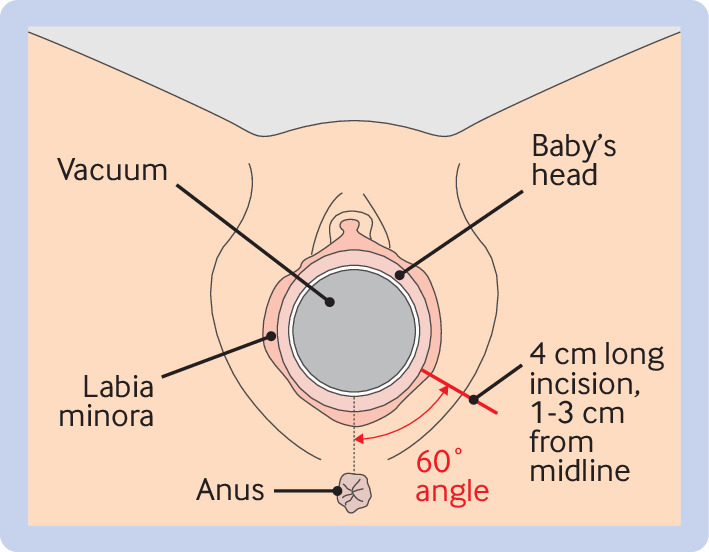
Illustration of a standardised lateral episiotomy in the EVA trial

In all women, the vacuum extraction procedure was prepared according to clinical routine, including intermittent bladder catheterisation, and ensuring pain relief in the form of epidural, pudendal, or local anaesthesia, or a combination of these. The extraction was done by the attending physician synchronously with maternal contractions and pushing until the fetal head was crowning, when a lateral episiotomy was done by the physician or midwife. The trial intervention was a standardised lateral episiotomy, beginning 1-3 cm from the posterior fourchette, at a 60° (45-80°) angle from the midline, and 4 cm (3-5 cm) long (fig 1), based on consensus^16^ and suggested protective trigonometric properties.^17^ ^18^ The clinical staff at all sites received education on several occasions on how to perform the standardised lateral episiotomy before trial start. The comparison was no episiotomy unless considered indispensable. All women received verbal guidance and manual perineal support to prevent obstetric anal sphincter injury according to clinical routine, including intended slow delivery of the fetal head, hands-on perineal support, and warm compresses. Examination and suturing of vaginal and perineal injuries were managed according to clinical routine. Postnatal care was supplied according to clinical routine. In addition, perineal pain was assessed once between day one and seven after childbirth using a numerical rating scale (from 0=no pain to 10=worst possible pain) and a questionnaire covering complications was sent out at two months after childbirth (supplementary table S3).

### Outcomes

The primary outcome was obstetric anal sphincter injury, defined as a third or fourth degree perineal injury involving the external or internal anal sphincter muscles, or both, as defined by the diagnoses O702 and O703, requiring surgical repair, in the Swedish version of the International Classification of Diseases 10th edition. Obstetric anal sphincter injury was the primary outcome in the published meta-analyses,^7^ ^8^ and carries well known risks of both short and long term pelvic floor sequelae.^24^ Obstetric anal sphincter injury rate is also an established marker of quality of care, comparable between hospitals and countries.^4^ ^6^ ^25^ The diagnosis was made clinically by the attending physician on site through visual inspection and digital vaginal and rectal examination immediately after childbirth, according to Swedish guidelines.^15^ Exploratory maternal outcomes were vaginal or perineal injury other than obstetric anal sphincter injury (ie, intact perineum, first degree injury, and second degree perineal injury including episiotomy, allocated or not), duration of hospital stay (days), perineal pain (once between days one and seven after delivery reported on a numerical rating scale from 0=no pain to 10=worst imaginable pain), and birth experience (once between days one and seven after delivery reported on a numerical rating scale from 1=worst possible overall experience to 10=best possible overall experience). Perineal pain of 7 or more was used as an arbitrary indicator of severe pain. Childbirth experience of 3 or less was used as an indicator of a negative childbirth experience.^26^ From the two month questionnaire, questions regarding perineal pain, surgical wound problems, infections, and re-admission were included (supplementary material). Secondary outcomes are patient reported outcomes collected at 12 months, with results anticipated in 2025.^21^

Exploratory neonatal outcomes were an Apgar score of less than 7 at 5 mins, metabolic acidosis defined as umbilical artery pH of less than 7.05 or base deficit of 12 mmol/L or more, admission to neonatal intensive care, shoulder dystocia, scalp haematoma, fetal fracture, obstetric brachial plexus palsy, hypoxic ischaemic encephalopathy, and neonatal seizures. Safety maternal outcomes were postpartum haemorrhage (blood loss of ≥1000 mL) and severe perineal pain (numerical rating scale of ≥7, measured once between days one and seven).

Demographics, maternal and childbirth characteristics, and outcomes were retrieved from the Swedish pregnancy register.^27^ Data not available in the register were registered in an electronic case report form for each participant. Second stage duration was defined as the time between full cervical dilatation and birth. Neonatal data were retrieved from the Swedish pregnancy register and the Swedish neonatal quality register.^28^ Data for vaginal and perineal injuries were registered in the electronic case report form. The primary outcome of obstetric anal sphincter injury was cross checked with data from the Swedish pregnancy register. Two individuals had discrepant results. In both cases, an erroneous procedure code in the Swedish pregnancy register did not match the diagnostic codes or text in the medical record. Therefore, the outcome obstetric anal sphincter injury was based on the electronic case report form.

### Adverse and serious adverse events

An adverse event was defined as a complication to the trial intervention or perineal injury including perineal wound infection, dehiscence, granuloma or symptomatic scarring, severe perineal pain (requiring opioids), or fistula formation in the vagina, anus, or perineum during the first eight weeks postpartum. A serious adverse event was any event resulting in maternal death within 42 days postpartum or neonatal death within 28 days after birth, or that was life threatening, required admission to intensive care unit, or that resulted in persistent or significant disability. Participants were instructed to contact the hospital where they gave birth in case of symptoms of adverse events. Adverse events were thus identified through self-referral. In addition to this, medical records were screened for adverse events up to two months after childbirth and self-reported complications were collected from the questionnaire at two months.

### Statistical analyses

The original sample size was based on the meta-analysis by Lund and colleagues,^8^ reporting a 50% reduction of obstetric anal sphincter injury in vacuum extraction in nulliparous women, when a lateral or mediolateral episiotomy was performed. We used mean rate of obstetric anal sphincter injury in vacuum extraction in Sweden in 2015, according to the Swedish medical birth register, to hypothesise that a 50% reduction of obstetric anal sphincter injury from 12.4% to 6.2% could be detected with 80% power and a P<0.05 with 344 women in each group using a two sided χ^2^ test. With an estimated 3% loss to follow-up, 355 women in each group (total n=710) was needed. After the Swedish network for national clinical studies in obstetrics and gynaecology (www.snaks.se) advised that a smaller difference could be clinically important, we applied for ethical approval for an amendment in 2017, after the trial had started, to include 1400 women to show a 30% reduction from 12.4% to 7.8%. In 2019, we aimed for the initial sample size (710 women) due to slow recruitment. An interim analysis was done in 2020 according to prespecified criteria to detect a difference in line with van Bavel and colleagues,^21^ ^29^ reporting a reduction from 14.0% to 2.5% of obstetric anal sphincter injury with episiotomy in nulliparous women requiring vacuum extraction. To show this difference with 80% power and P<0.01, 350 women were needed, a total which was attained in 2020. In case this difference was found, we had a priori decided to stop the trial. Stopping criteria were not met.

**Fig 2:**
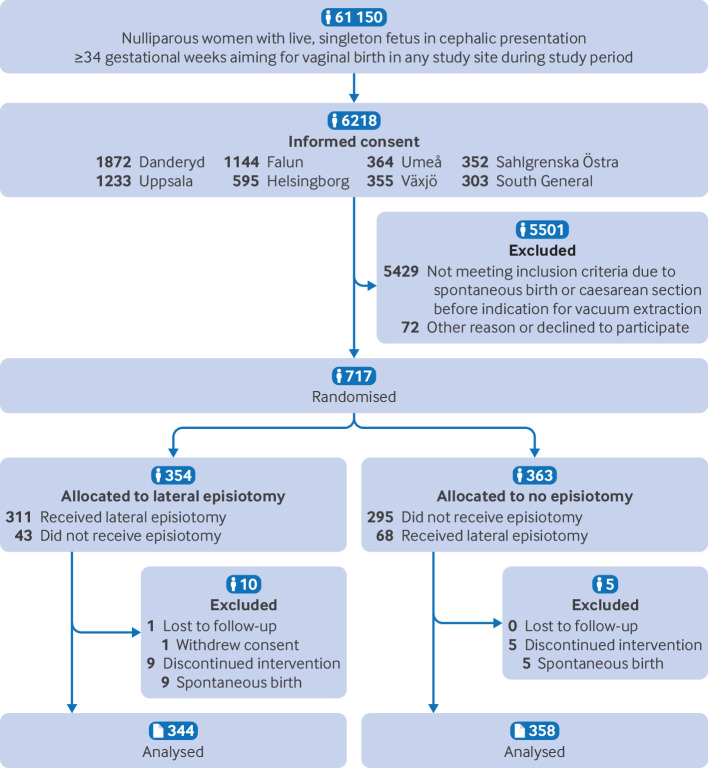
CONSORT flowchart of the modified intention-to-treat population

For the main comparative analyses, we used a modified intention-to-treat population, defined as all consenting randomised women with vacuum extraction or vacuum extraction attempt. A vacuum extraction attempt was defined as having the vacuum cup applied to the fetal head. One woman withdrew consent before the vacuum cup was applied and was excluded. Women who had been randomised but had a spontaneous birth before the vacuum cup was applied were also excluded (fig 2). This modification to the intention-to-treat population was motivated by the primary objective of the trial, because women with spontaneous birth were deemed to have lost the main inclusion criterium (vacuum assisted delivery). The primary analysis was based on allocation regardless of received treatment. We also analysed the outcome of obstetric anal sphincter injury on the total intention-to-treat (all consenting women), per protocol (consenting women delivered with vacuum extraction who received the assigned treatment), as treated (consenting women with attempted or successful vacuum extraction), and safety populations (all consenting women as treated).

Baseline characteristics are presented with medians and ranges or numbers and proportions. The prespecified primary efficacy analysis was the unadjusted comparison of the primary outcome obstetric anal sphincter injury in the lateral episiotomy group compared with the no episiotomy group on the modified intention-to-treat population with a two sided χ^2^ test. Due to the performed interim analysis, P<0.04 was deemed necessary for the primary outcome of obstetric anal sphincter injury to account for multiple testing. The risk difference and risk ratio between the groups were calculated with 96% CI using the method of Miettinen and Nurminen.^30^ Risk differences of less than 0 and risk ratios of less than 1 indicated lower risks for the outcome in the intervention group versus the control group. Since the randomisation was stratified by site, adjustment for site effects was done by using a mixed effects Poisson regression model to estimate the risk ratio with 96% CI, with site and site × treatment as random effects. The interaction term was included to account for treatment effect heterogeneity across sites. Site specific treatment effects were calculated using the best linear unbiased predictions of the random effects and visualised in a forest plot. In a prespecified sensitivity analysis, we also adjusted for maternal age, height, body mass index, country of birth, fetal position, and operator skills (categorised into resident, specialist gynaecologist, or specialist obstetrician), by inclusion as covariates in the mixed effects Poisson model. Number needed to treat were calculated with 96% CI and number needed to harm were calculated with 95% CI.

Maternal and neonatal categorical exploratory outcomes were compared with two sided χ^2^ test, or Fisher’s exact test for rare events, and t test for continuous variables. Maternal safety outcomes, including adverse events and serious adverse events, were calculated for the modified intention-to-treat population based on allocated treatment and for the safety population based on received treatment. All analyses except the primary outcome were unadjusted, P<0.05 was considered significant, and risk difference and risk ratio with 95% CI were calculated. A detailed statistical analysis plan was set before data lock on 27 June 2023 and is available as supplementary information. Changes from the protocol included the use of Poisson regression to estimate the relative risk instead of logistic regression to estimate the odds ratio. Analyses were performed by independent, endpoint masked statisticians using SAS 9.4 (SAS Institute Inc, Cary, NC, USA).

### Patient and public involvement

At the initiation of this trial, patient or public involvement was not a compulsory requirement and, at the time, no relevant patient organisations existed. Therefore, layperson patients or the public were not directly involved in the design or conduct of this trial. However, in 2015 the Swedish government commissioned the healthcare regions, research councils, and authorities to improve prevention of obstetric anal sphincter injury. The Swedish Agency for Health Technology Assessment and Assessment of Social Services (SBU), with the contribution of patient representatives, published a report in 2018 of prioritised research areas within the field of maternal birth injuries. The report stated that preventative measures against obstetric anal sphincter injury was one of the most important research questions.^31^ In addition, along the conduct of this trial, a qualitative research project assessed the views of consenting and non-consenting women regarding the recruitment process.^32^ To increase the impact of the results from this trial, both for care providers and researchers, as well as for patients and public, the results will be disseminated in conference presentations, posters, and mass media, and shared across social media, pregnancy podcasts, and companion blogs to involve pregnant women and their families.

## Results

During the recruitment period of 1 July 2017 to 15 February 2023, 61 150 women were eligible at the eight study sites, of which 6218 women consented to participate if vacuum extraction was required. Of these, a total of 717 women required vacuum extraction and were randomly assigned by 255 different physicians. In all, 354 (49%) women were allocated to lateral episiotomy and 363 (51%) women were allocated to no episiotomy (fig 2). One woman from the intervention group withdrew consent before the vacuum extraction and was excluded. Spontaneous birth occurred before the vacuum was applied in nine women allocated to lateral episiotomy and in five women allocated to no episiotomy. These women were also excluded from the modified intention-to-treat population. Therefore, the modified intention-to-treat population included 344 (49%) women allocated to lateral episiotomy and 358 (51%) women allocated to no episiotomy (fig 2).

Of the 344 women allocated to lateral episiotomy, 310 (90%) received a lateral episiotomy and 34 (10%) received no episiotomy. Of 358 women allocated to no episiotomy, 291 (81%) received no episiotomy, while 67 (19%) received a lateral episiotomy. The two allocation groups were similar regarding maternal (table 1) and childbirth characteristics (table 2). Among women allocated to lateral episiotomy, 22 (6%) were delivered by caesarean section after vacuum extraction was unsuccessful, of which four received an episiotomy before conversion. Among women allocated to no episiotomy, five (1%) were delivered by caesarean section after vacuum extraction was unsuccessful. None of these women received an episiotomy.

**Table 1:** Maternal demographic and baseline characteristics (modified intention-to-treat population)

Characteristics	Lateral episiotomy (n=344)	No episiotomy (n=358)
Age (years), median (range)	31 (19-43)	31 (21-47)
Age (years):		
<19	1 (<1)	0
20-24	22 (6)	20 (6)
25-29	100 (29)	115 (32)
30-34	149 (43)	134 (37)
≥35	72 (21)	89 (25)
Country of birth:		
Sweden	266 (77)	286 (80)
Other European	33 (10)	20 (6)
Outside Europe	23 (7)	26 (7)
Missing	22 (6)	26 (7)
Educational level:		
None or less than nine years	1 (<1)	2 (1)
Compulsory school (nine years)	7 (2)	11 (3)
Upper secondary school (gymnasium)	104 (30)	76 (21)
University/college	205 (60)	231 (65)
Missing	27 (8)	38 (11)
Height (cm):		
Median (range)	166 (150-185)	167 (147-182)
<160	34 (10)	44 (13)
Missing	4 (1)	7 (2)
Body mass index:		
Median (range)	23.7 (17.7-50.1)	23.7 (16.6-44.5)
≤18.4	6 (2)	8 (2)
18.5-24.9	207 (60)	207 (58)
25.0-29.9	88 (26)	99 (28)
30.0-4.9	23 (7)	20 (6)
≥35.0	11 (3)	15 (4)
Missing	9 (3)	9 (3)
Pregnancy complications:		
PROM/PPROM	50 (15)	60 (17)
Hypertensive disease/pre-eclampsia	33 (10)	38 (11)
Gestational and pregestational diabetes mellitus	20 (6)	26 (7)
Intrauterine growth restriction/oligohydramniosis	11 (3)	12 (3)
Accelerated growth/macrosomia	9 (3)	12 (3)
Study site:		
Uppsala University Hospital	92 (27)	92 (26)
Danderyd University Hospital	80 (23)	83 (23)
Falun Hospital	69 (20)	78 (22)
Helsingborg Hospital	37 (11)	33 (9)
Umeå University Hospital	15 (4)	20 (6)
South General Hospital	19 (6)	16 (5)
Östra Sahlgrenska University Hospital	14 (4)	19 (5)
Växjö Hospital	18 (5)	17 (5)

Data are number (percentage), unless otherwise specified.

PROM=prelabour rupture of membranes; PPROM=preterm, prelabour rupture of membranes.

**Table 2:** Childbirth characteristics (modified intention-to-treat population)

Childbirth characteristics	Lateral episiotomy (n=344)	No episiotomy (n=358)
Gestational age at randomisation:		
Days, median (range)	283 (249-296)	284 (250-296)
≥40 weeks	226 (66)	247 (69)
Mode of onset:		
Induction	133 (39)	154 (43)
Spontaneous	211 (61)	204 (57)
Epidural anaesthesia	312 (91)	324 (91)
Oxytocin augmentation	334 (97)	346 (97)
Second stage duration:		
hh:min, median (range)	3:41 (00:06-10:07)	3:39 (00:06-08:46)
Indication for vacuum extraction*:		
Fetal distress	156 (45)	169 (47)
Labour dystocia/no progression	174 (51)	164 (46)
Maternal exhaustion	68 (20)	77 (22)
Correction of fetal position	3 (1)	3 (1)
Other	0	4 (1)
Fetal station:		
At ischial spines	7 (2)	8 (2)
Below ischial spines	89 (26)	86 (24)
Above pelvic floor	177 (52)	199 (56)
At pelvic floor	71 (21)	65 (18)
Fetal position:		
Occiput anterior	288 (84)	307 (86)
Occiput posterior	28 (8)	31 (9)
Other	8 (2)	6 (2)
Missing	8 (2)	6 (2)
Operator skills:		
Resident	133 (39)	154 (43)
Specialist Gynaecologist	69 (20)	66 (18)
Specialist Obstetrician	142 (41)	138 (39)
No of pulls:		
<6	316 (92)	327 (91)
≥6	27 (8)	31 (9)
Missing	1 (<1)	0
Vacuum cup detachment, any	37 (11)	30 (8)
Conversion to caesarean section	22 (6)	5 (1)
Birthweight (g):		
Median (range)	3565 (2260-5025)	3580 (2438-4850)
Mean (SD)	3573 (461)	3591 (454)
≥4000	60 (17)	59 (17)
Head circumference (cm):		
Median (range)	36 (31-40)	35 (31-44)
Mean (SD)	35.4 (2.2)	35.5 (1.6)
≥38	4 (1)	9 (3)

Data are number (percentage), unless otherwise specified.

* More than one indication optional.

Obstetric anal sphincter injury occurred in 21 (6%) of the women allocated to lateral episiotomy and in 47 (13%) of the women allocated to no episiotomy (P=0.002), with a risk difference of −7.0% (96% CI −11.7% to −2.5%) and risk ratio of 0.46 (96% CI 0.28 to 0.78). The adjusted risk ratio (adjusted for study site) was 0.47 (96% CI 0.23 to 0.97) (table 3). Results were largely consistent across sites, although the crude event rates were numerically larger in the episiotomy group for two of eight sites (supplementary figure S1). Similar results were also observed in a prespecified sensitivity analysis adjusting for maternal age, height, body mass index, country of birth, fetal position, and operator skills (adjusted risk ratio 0.49 (96% CI 0.24 to 0.99)). Number needed to treat with episiotomy was 14.3 (96% CI 8.6 to 40.0) to avoid one obstetric anal sphincter injury. In the per protocol population, obstetric anal sphincter injury occurred in 20 (6%) of 311 women who received lateral episiotomy and 38 (13%) of 295 women with no episiotomy (risk difference −6.5% (95% CI −11.4% to −1.8%); risk ratio 0.50 (95% CI 0.30 to 0.84)). Obstetric anal sphincter injury for the total intention-to-treat population had consistent results with the modified intention-to-treat and per protocol analyses. In the as treated population, obstetric anal sphincter injury occurred in 29 (8%) of 377 women with lateral episiotomy and 39 (12%) of 325 women with no episiotomy, which did not reach statistical significance (risk difference −4.3% (95% CI −8.9% to 0.2%)) (supplementary table S1).

**Table 3:** Primary and exploratory outcomes (modified intention-to-treat population)

Outcomes	Lateral episiotomy, no (%) (n=344)	No episiotomy, no (%) (n=358)	P value	Risk difference, %*	Risk ratio*
**Primary outcome**					
OASI	21 (6)	47 (13)	0.002	−7.0 (−11.7 to −2.5)	0.46 (0.28 to 0.78)
OASI adjusted†	-	-	0.031	—	0.47 (0.23 to 0.97)
**Exploratory outcomes, maternal**					
Intact perineum	12 (4)	6 (2)	0.16	1.8 (−0.6 to 4.5)	2.08 (0.79 to 5.48)
Caesarean section	12	4	—	—	—
First degree injury	8 (2)	46 (13)	<0.001	−10.5 (−14.6 to −6.7)	0.18 (0.09 to 0.38)
Caesarean section	5	1	—	—	—
Second degree injury or episiotomy	303 (88)	259 (72)	<0.001	15.7 (9.9 to 21.4)	1.22 (1.13 to 1.31)
Caesarean section	5	0	—	—	—
Postpartum haemorrhage ≥1000 mL	51 (17)	60 (19)	0.54	−1.8 (−7.8 to 4.1)	0.90 (0.64 to 1.26)
Postpartum haemorrhage (mL), mean (SD)	647 (436)	654 (510)	0.86	—	—
Missing	37	33	—	—	—
Perineal pain‡, NRS ≥7	144 (49)	142 (45)	0.44	3.1 (−4.8 to 11.0)	1.07 (0.90 to 1.27)
Pain‡, median (range)	6 (1-10)	6 (1-10)	0.20	—	—
Missing	47	45	—	—	—
Birth experience‡, NRS ≤3	32 (12)	42 (14)	0.32	−2.8 (−8.3 to 2.8)	0.81 (0.52 to 1.24)
Birth experience‡, median (range)	7 (1-10)	7 (1-10)	0.39	—	—
Missing	66	64	—	—	—
Hospital stay ≥3 days	64 (19)	76 (22)	0.37	−2.8 (−8.7 to 3.2)	0.87 (0.65 to 1.17)
Hospital stay, days, mean (SD)	2.25 (1.22)	2.36 (1.32)	0.23	—	—
Missing	5	7	—	—	—
**Exploratory outcomes, neonatal**					
Apgar <7 at 5 min	6 (2)	12 (3)	0.18	−1.6 (−4.3 to 0.8)	0.52 (0.20 to 1.37)
Missing	0	1	-	-	-
Metabolic acidosis§	22 (9)	26 (10)	0.69	−1.1 (−6.5 to 4.4)	0.90 (0.52 to 1.54)
Missing	108	108	-	-	-
NICU admission	31 (9)	34 (10)	0.61	−0.5 (−4.8 to 3.9)	0.94 (0.60 to 1.51)
Shoulder dystocia	0	3 (1)	0.09	−0.8 (−2.4 to 0.3)	n/a
Scalp haematoma	19 (6)	11 (3)	0.11	2.4 (−0.6 to 5.7)	1.79 (0.87 to 3.71)
Fracture	3 (1)	1 (<1)	0.30	0.6 (−0.8 to 2.3)	3.11 (0.33 to 29.79)
OBPP	1 (<1)	0	0.31	0.3 (−0.8 to 1.6)	n/a
HIE	1 (<1)	1 (<1)	0.98	0.3 (−0.8 to 1.6)	1.04 (0.07 to 16.57)
Seizures	1 (<1)	2 (1)	0.59	−0.3 (−1.5 to 1.0)	0.52 (0.05 to 5.71)

CI=confidence interval; HIE=hypoxic ischaemic encephalopathy; NICU=neonatal intensive care unit; NRS=numerical rating scale (1-10); OASI=obstetric anal sphincter injury; OBPP=obstetric brachial plexus palsy; SD=standard deviation.

* Primary outcome had 96% confidence intervals and exploratory outcomes had 95% confidence intervals.

† Adjusted for study site.

‡ Pain and birth experience were assessed by numerical rating scales in the postnatal ward (pain 0-10, where 0=no pain to 10=worst imaginable pain and birth experience 1-10, where 1=worst possible overall experience and 10=best possible overall experience).

§ Metabolic acidosis is defined as umbilical artery pH <7.05 or base deficit 12.0 mmol/L or more.

Intact perineum was rare and most of these participants gave birth by caesarean section. In the intervention group, first degree injuries were significantly less frequent and second degree injuries including episiotomy (allocated or not) were significantly more frequent, compared with the comparison group. No statistically significant differences were noted for postpartum haemorrhage, postpartum perineal pain, birth experience, hospital stay, or neonatal outcomes (table 3).

Self-referral of wound infection and dehiscence were significantly more common in the intervention group than in the control group. Number needed to harm was 21.7 (95% CI 12.5 to 125) for infection and 16.9 (10.2 to 41.7) for wound dehiscence. Total self-referred adverse events did not differ significantly between the groups and no significant difference was noted in surgical treatment such as wound re-suturing or extirpation of granuloma. Serious adverse events and persistent incapacity were rare and did not differ significantly between groups (table 4). The differences in wound complications increased in the safety population (supplementary table S2).

**Table 4:** Adverse events and serious adverse events (self-referral) (modified intention-to-treat population)

Outcomes	Lateral episiotomy, no (%) (n=344)	No episiotomy, no (%) (n=358)	P value	Risk difference, % (95% CI)	Risk ratio (95% CI)
Adverse events					
Wound infection	32 (9)	17 (5)	0.02	4.6 (0.8 to 8.5)	1.96 (1.11 to 3.46)
Wound dehiscence	32 (9)	12 (3)	0.001	6.0 (2.4 to 9.8)	2.78 (1.45 to 5.30)
Granuloma or scarring	14 (4)	21 (6)	0.27	−1.8 (−5.2 to 1.5)	0.69 (0.35 to 1.34)
Surgical treatment*	25 (7)	20 (6)	0.36	1.7 (−2.0 to 5.5)	1.30 (0.74 to 2.30)
Severe pain	21 (6)	26 (7)	0.54	−1.2 (−4.9 to 2.6)	0.84 (0.48 to 1.47)
Fistula formation	1 (<1)	0	0.31	0.3 (−0.8 to 1.6)	N/A
Any of the above	74 (22)	62 (17)	0.16	4.2 (−1.7 to 10.0)	1.24 (0.92 to 1.68)
**Serious adverse events **					
Maternal death between days 0-42	0	0	N/A	0	N/A
Maternal critical care†	1 (<1)	1 (<1)	0.98	0.0 (−1.3 to 1.4)	1.04 (0.07 to 16.57)
Persistent incapacity‡	4 (1)	1 (<1)	0.37	0.9 (−0.6 to 2.7)	4.16 (0.47 to 37.06)
Neonatal death between days 0-28	0	0	N/A	0	N/A

N/A=not applicable.

* Including re-suturing of wound or extirpation of granuloma.

† One woman who required intensive care due to extreme blood loss and deranged coagulation because she refused to take blood for religious reasons; and one woman with septicaemia.

‡ Persistent incapacity is defined as still ongoing after one year or with sequelae and were in these five cases one woman who required intensive care due to extreme blood loss and deranged coagulation because she refused to take blood for religious reasons; one woman with fistula formation; and two women with wound dehiscence; and one with granuloma that the site principal investigator deemed had recovered but with sequelae.

For self-reported complications within two months after childbirth, no significant difference in pain assessment was noted but women in the intervention group significantly more often used analgesics after discharge from the hospital. No significant difference was noted in duration of analgesics use. Similar to the results from self-referral, the intervention group reported more wound complications, including wound infection and wound re-suturing (table 5).

**Table 5:** Self-reported complications within two months after childbirth (modified intention-to-treat population)

Complications	Lateral episiotomy(n=252)	No episiotomy (n=245)	P value	Risk difference, % (95% CI)	Risk ratio (95% CI)
Pain, NRS, mean (SD)	2.3 (1.7)	2.2 (1.6)	0.82	—	—
Pain, NRS ≥7	10 (4)	7 (3)	0.50	1.1 (−2.3 to 4.6)	1.39 (0.54 to 3.59)
Analgesics*					
Analgesics, any use	215 (85)	178 (73)	<0.001	12.7 (5.5 to 19.7)	1.17 (1.07 to 1.29)
Days, mean (SD)	14.7 (9.0)	13.7 (8.3)	0.26	—	—
Duration ≥7 days	146 (68)	120 (67)	0.92	0.5 (−8.7 to 9.8)	1.01 (0.88 to 1.16)
Wound complication†	75 (30)†	43 (18)§	0.001	12.2 (4.7 to 19.5)	1.70 (1.22 to 2.36)
Caesarean section	3	1	—	—	—
Re-suturing	14 (6)	3 (1)	0.01	4.3 (1.1 to 8.0)	4.54 (1.32 to 15.59)
Fistula formation	1 (<1)	0	0.19	0.3 (−0.8 to 1.6)	—
Infection, any of below	42 (17)	34 (14)	0.39	2.8 (−3.6 to 9.1)	1.20 (0.79 to 1.82)
Urinary tract infection	5 (2)	9 (4)	0.25	−1.7 (−5.0 to 1.4)	0.54 (0.18 to 1.58)
Genital infection	9 (4)	12 (5)	0.45	−1.3 (−5.2 to 2.4)	0.72 (0.31 to 1.69)
Wound infection	24 (9)	11 (5)	0.03	5.0 (0.5 to 9.7)	2.11 (1.05 to 4.20)
Endometritis	5 (2)	10 (4)	0.17	−2.1 (−5.6 to 1.1)	0.48 (0.17 to 1.39)
Sepsis	1 (<1)	1 (<1)	0.98	0 (−1.9 to 1.8)	0.97 (0.06 to 15.34)
Other infection	7 (3)	8 (3)	0.74	−0.5 (−3.8 to 2.8)	0.84 (0.31 to 2.29)
Re-admission	27 (11)	29 (12)	0.67	−1.1 (−6.7 to 4.4)	0.90 (0.55 to 1.47)

Data are number (percentage), unless otherwise specified. Unadjusted risk difference and risk ratio are presented.

NRS=numerical rating scale (1-10).

* After discharge.

† Wound complication was defined as an affirmative answer to “describe your discomfort/complication by choosing the area/areas affected”: ticked box “wound” (supplementary material).

## Discussion

### Principal findings

In this randomised trial of 702 nulliparous women requiring vacuum extraction, the rate and risk of obstetric anal sphincter injury was more than halved in women allocated to lateral episiotomy compared with no episiotomy. No significant differences were noted in blood loss, perineal pain, birth experience, hospital stay duration, short term neonatal outcomes, or total adverse events. Women allocated to lateral episiotomy had more wound infections, dehiscence, and re-suturing, but when including extirpation of granulomas, surgical treatment did not significantly differ between groups.

### Strengths and limitations

This study is the first adequately sized randomised controlled trial to assess the effect of episiotomy in nulliparous women requiring vacuum extraction, filling the previous knowledge gap.^6^ ^8^ ^13^ Other strengths of this trial are the small differences between results in the modified intention-to-treat, intention-to-treat, per protocol, and as treated analyses, the excellent protocol adherence, and the representative population and staff from eight hospitals evenly distributed over Sweden.

A limitation of this trial is that the obstetric anal sphincter injury diagnosis was not masked for the allocation because this was deemed impossible as an episiotomy would be apparent. Instead, the sites followed Swedish guidelines recommending a joint assessment of two care providers when diagnosing perineal injuries.^15^ One of these providers was often the same physician who performed the vacuum extraction. This reflects clinical practice but could infer a risk of detection bias, especially if the provider had a strong opinion of the effect of episiotomy. Since vacuum extraction was performed unplanned at all hours, an independent investigator was not deemed feasible. Ultrasound immediately after childbirth to objectively assess obstetric anal sphincter injury was neither considered feasible,^33^ nor is it recommended.^34^ Nevertheless, to check the diagnostic accuracy, as is recommended in the postnatal period in high risk births,^34^ an ongoing substudy blinded for allocation in four sites using 3D endoanal and endovaginal ultrasound at 6-12 months postpartum is expected to have results in 2025.^21^

### Comparison with other studies

Two previous randomised controlled trials have assessed the effect of episiotomy in operative vaginal delivery.^19^ ^20^ Neither of these was adequately sized to confirm or refute an effect. The level of risk reduction and number needed to treat in our trial confirm the results of pooled observational studies.^7^ ^8^ Nevertheless, the confidence interval for number needed to treat was wide, which may be due to a limited sample size or a variation in effect also seen in observational studies.^8^ ^24^ The average number needed to treat may be difficult to interpret because the average does not refer to a particular individual, which may carry a higher or lower baseline risk of obstetric anal sphincter injury. However, the average number needed to treat should be acceptable also in settings where episiotomy is used restrictively.^35^ The rate of obstetric anal sphincter injury in the total study population is also consistent with the current rate among Swedish primiparous women delivered with vacuum extraction.^36^

Episiotomy has been associated with greater blood loss and perineal pain,^13^ although this was not confirmed in our trial. Likewise, no clear evidence suggests that lateral episiotomy significantly affected birth experience, hospital stay duration, or short term neonatal outcomes. However, the confidence intervals were wide which makes equipoise for these outcomes uncertain. Women allocated to episiotomy reported more wound infection, coherent with previous observational studies.^37^ ^38^ This complication might have been prevented if prophylactic antibiotics had been given, as shown in the ANODE trial from 2019.^38^ During our trial, Swedish guidelines recommended prophylactic antibiotics only for obstetric anal sphincter injury,^15^ which potentially could have imbalanced wound complications in the groups. Given the results from the ANODE trial and our trial, prophylactic antibiotics should be given in vacuum extraction.^38^

The major objection against routine episiotomy is the risk of an unnecessary cut, perhaps causing a larger injury than needed. Our study supports that routine lateral episiotomy will result in a higher proportion of women sustaining a second degree injury, instead of a first degree injury or obstetric anal sphincter injury. The proportion of women with intact perineum was similar in both groups, partly due to more conversions to caesarean section in the intervention group. Conversion to caesarean section was in most cases done before the intervention, therefore, this difference was deemed to result from chance. It is also not plausible that episiotomy would increase the risk of failed vacuum extraction.

A potential pitfall in this trial is the uneven non-adherence to allocated treatment, which was more common in the no episiotomy group. Episiotomy was in these cases usually motivated by fetal distress or imminent tearing, as restrictive practice implies. In comparison with previous trials,^19^ ^20^ the protocol adherence in the no episiotomy group was excellent. Notably, the clinical judgement of who did and did not need an episiotomy did not improve the reduction of obstetric anal sphincter injury as seen in the as treated analysis. This effect is consistent with a previous meta-analysis reporting that an episiotomy rate of over 75% had a greater protective effect.^8^ Admittedly, it seems possible to maintain a low rate of episiotomy and a low rate of obstetric anal sphincter injury in some settings,^20^ which suggests that other factors, such as operator skills, may contribute.^39^

### Conclusions and policy implications

Until now, guidelines have advised clinicians to consider episiotomy in nulliparous women requiring vacuum extraction, without a clear recommendation. With the results of our study, a lateral episiotomy can be recommended for nulliparous women requiring vacuum extraction to significantly lower the risk of obstetric anal sphincter injury. However, before recommending routine lateral episiotomy in nulliparous women requiring vacuum extraction, more evidence is needed regarding long term patient reported outcomes. For example, outcomes such as quality of life and pelvic floor function after vacuum extraction with and without lateral episiotomy. These outcomes will be assessed in the planned follow-up of the EVA trial. The follow-up results can inform the design of an optimal prophylactic strategy.

**What is already known on this topic**

Obstetric anal sphincter injury is a serious complication to vaginal birth, leading to anal incontinence and reduced quality of life, and is more common in nulliparous women delivered with vacuum extraction
Lateral or mediolateral episiotomy might reduce obstetric anal sphincter injury in nulliparous women delivered with vacuum extraction by approximately 50%
The effect of a lateral or mediolateral episiotomy in instrumental births in nulliparous women has been called on to be investigated in an adequately sized randomised controlled trial

**What this study adds**

This trial provides evidence that the rate of obstetric anal sphincter injury in nulliparous women requiring vacuum extraction can be significantly reduced with a lateral episiotomy
No differences were noted between groups in postpartum pain, blood loss, neonatal outcomes, or total adverse events, but episiotomy was significantly associated with an increase in wound infection and dehiscence
Lateral episiotomy can be recommended in nulliparous women requiring vacuum extraction to reduce the rate of obstetric anal sphincter injury

## Data Availability

The study protocol and the statistical analysis plan is available with publication as supplementary material. Deidentified individual participant data and a data dictionary defining each field in the set will be made available to others for meta-analysis with investigator support after approval of a proposal (sophia.brismar-wendel@regionstockholm.se) and with a signed data access agreement.

## Web extra

Extra material supplied by authors

Web appendix: Extra material supplied by authors

## Notes

Contributors: SBW conceived the research idea and led the trial. SBW, HKK, MJ, and SH attained funding for the trial. All authors were principal investigators or investigators and contributed to the completion of the trial. SB, MJ, SH, and SBW are responsible for the statistical analysis plan. SB, VA, MJ, SH, ACW, ER, ÅL, HF, and SBW checked the original data. SB and SBW directly accessed and verified the underlying data and analyses. SB wrote the first draft of the manuscript with input from MJ, SH, ÅL, HKK, and SBW. All authors approved of the submitted version. The corresponding author attests that all listed authors meet authorship criteria and that no others meeting the criteria have been omitted. SBW acts as a guarantor.

Transparency: SBW affirms that the manuscript is an honest, accurate, and transparent account of the study being reported; that no important aspects of the study have been omitted; and that any discrepancies from the study as planned and registered have been explained.

Dissemination to participants and related patient and public communities: The results were partly presented for the first time on 28 August 2023 in Trondheim, Norway, at a meeting organised by the Nordic Federation of Obstetrics and Gynaecology, to invited laypersons, obstetricians, gynaecologists, and midwives. The results were partly presented a second time on 11 October 2023 in Paris, France, at a congress organised by the International Federation of Gynaecology and Obstetrics, to invited patient association representatives as well as decision makers, obstetricians, gynaecologists, and midwives. We plan to disseminate results to the public through the media departments and websites of the authors’ institutions, and by sharing the results across social media, pregnancy podcasts, and companion blogs to involve pregnant women and their families in discussing the implications and further research.

Provenance and peer review: Not commissioned; externally peer reviewed.
