# Supplementary material for: Lateral episiotomy or no episiotomy in vacuum assisted delivery in nulliparous women (EVA): multicentre, open label, randomised controlled trial
Source: BMJ. 2024 Jun 17;385:e079014. doi: 10.1136/bmj-2023-079014 (PMC11307825; doi:10.1136/bmj-2023-079014)
Supplement: Supplementary file 1 — Web appendix: Extra material supplied by authors [file bers079014.ww.pdf]

Supplementary Figure S1. Risk ratio (RR) of obstetric anal sphincter injury with lateral episiotomy vs no episiotomy on the mITT population, by study site.

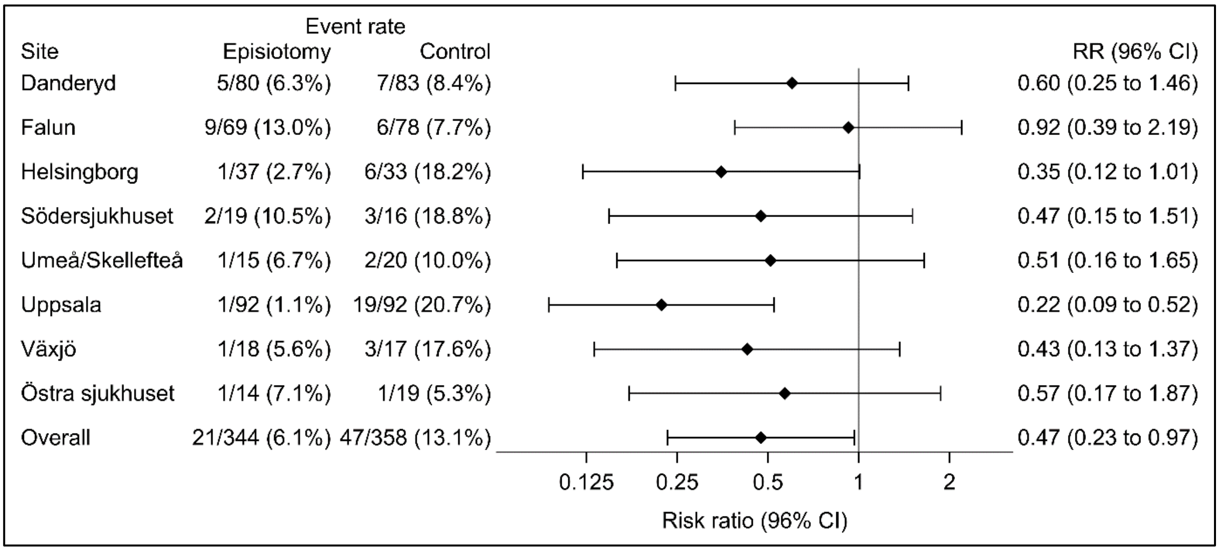

Statistical analyses were performed using a mixed effects Poisson regression model with site and treatment x site as random effects to account for site effects and treatment effect heterogeneity across sites. Site-specific effects were estimated using the best linear unbiased predictions of the random effects from the mixed effects model.

Supplementary Table S1. Obstetric anal sphincter injury by population definition.

	Lateral episiotomy n (%)	No episiotomy n (%)	*P*	Risk difference	Risk ratio
Modified intention-to-treat	n=344	n=358		**(96% CI)**	**(96% CI)**
	21 (6.1)	47 (13.1)	0.002	-7.0 (-11.7; -2.5)	0.46 (0.28; 0.78)
					0.47 (0.23; 0.97)*
Intention-to-treat	n=353	n=363		**(95% CI)**	**(95% CI)**
	21 (5.9)	47 (12.9)	0.001	-7.0 (-11.4; -2.8)	0.46 (0.28; 0.75)
					0.47 (0.24; 0.92)*
Per-protocol	n=311	n=295			
	20 (6.4)	38 (12.9)	0.007	-6.5 (-11.4; -1.8)	0.50 (0.30; 0.84)
As-treated	n=377	n=325			
	29 (7.7)	39 (12.0)	0.054	-4.3 (-8.9; 0.2)	0.64 (0.41; 1.01)

Modified intention-to-treat population: All randomised women who had the vacuum cup applied, except those who withdrew their consent after randomisation and before intervention and primary outcome and except those who gave birth before the vacuum cup was applied, analysed by allocated treatment.
Intention-to-treat population: All randomised women, except those who withdrew their consent after randomisation and before intervention and primary outcome, regardless of mode of delivery, analysed by allocated treatment.
*Adjusted for study site
Per-protocol population: All randomised women who gave birth vaginally after the vacuum cup was applied, and who received the allocated treatment, except those who withdrew their consent after randomization and before intervention and primary outcome, analysed by allocated = received treatment.
As-treated population: The same women as in the modified intention-to-treat population but analysed by received treatment.

Supplementary Table S2. Adverse events in the safety population including all randomized women analysed as treated.

	Lateral episiotomy n (%) n=378	No episiotomy n (%) n=338	*P*	Risk difference (95% CI)	Risk ratio (95% CI)
**Adverse events**					
Wound infection	36 (9.5)	13 (3.8)	0.003	5.7 (2.0; 9.4)	2.48 (1.34; 4.59)
Wound dehiscence	38 (10.1)	6 (1.8)	<0.001	8.3 (4.9; 11.9)	5.66 (2.42; 12.23)
Granuloma or scarring	18 (4.8)	17 (5.0)	0.87	-0.3 (-3.6; 3.0)	0.95 (0.50; 1.81)
Surgical treatment*	32 (8.5)	13 (3.8)	0.01	4.6 (1.1; 8.2)	2.20 (1.18; 4.12)
Severe pain (opioids)	28 (7.4)	19 (5.6)	0.34	1.8 (-1.9; 5.5)	1.32 (0.75; 2.32)
Fistula formation	1 (0.3)	0	1.00	0.3 (-0.9; 1.5)	n/a
Any of the above	93 (24.6)	43 (12.7)	<0.001	11.9 (6.2; 17.4)	1.93 (1.39; 2.69)
**Serious adverse events**					
Maternal death day 0-42	0	0		0	
Maternal critical care†	2 (0.5)	0	0.50	0.5 (-0.7; 1.9)	n/a
Persistent incapacity‡	3 (0.8)	2 (0.6)	1.00	0.2 (-1.4; 1.8)	1.34 (0.23; 7.98)
Neonatal death day 0-28	0	0	n/a	0	n/a

*Including re-suturing of wound or extirpation of granuloma.

† One woman who required intensive care due to extreme blood loss, deranged coagulation and refusing to take blood for religious reasons, and one woman with septicaemia.

‡ Persistent incapacity is defined as still ongoing after one year or with sequels: one woman who required intensive care due to extreme blood loss, deranged coagulation and refusing to take blood for religious reasons, one woman with fistula formation, and two women with wound dehiscence, and one with granuloma that the site principal investigator deemed had recovered but with sequels.

Supplementary Material. Included questions from the two months questionnaire.

1. If you have any pain in the genital area, how would you rate it? (0= no pain, 10= extreme pain)

2. Did you stay in hospital after the delivery?
   1) No, I left the hospital the same day as the delivery
   2) Yes, I was admitted for at least one night after the delivery

3. How many nights did you stay in hospital after the delivery?

4. Have you had to take pain relief due to the operation since leaving the hospital?
   1) No 2) Yes

5. For how many days have you had to take pain relief due to the operation since leaving the hospital?

6. Describe your discomfort/complication by choosing the area/areas affected
   1) Wound (included)

7. Did the complication cause any of the following issues?
   1) Rupture of the wound scar requiring further operation
   2) False passage (fistula) between the vagina and bowel or bladder
   3) None of the above

8. Did you experience an infection of any of the following organs:
   1) Urinary tract infection
   2) Genital infection, offensive discharge
   3) Wound infection
   4) Infection in the womb
   5) Sepsis
   6) Other infection
   7) (NA) No infection

9. Were you admitted to hospital due to the complication?
   1) No, left the same day
   2) Yes, admitted one night
   3) Yes, admitted for two or more nights

10. Did you require an operation as part of the treatment?
    1) No 2) Yes
